# Substantial parallel mediation contribution by cognitive domains in the relationship between adolescents’ physical fitness and academic achievements: the Cogni-Action Project

**DOI:** 10.3389/fpsyg.2024.1355434

**Published:** 2024-07-10

**Authors:** Carlos Cristi-Montero, Ricardo Martínez-Flores, Juan Pablo Espinoza-Puelles, Anya Doherty, Juan Pablo Zavala-Crichton, Nicolas Aguilar-Farias, Tomas Reyes-Amigo, Vanessa Salvatierra-Calderon, Romualdo Ibáñez, Kabir P. Sadarangani

**Affiliations:** ^1^IRyS Group, Physical Education School, Pontificia Universidad Católica de Valparaíso, Valparaíso, Chile; ^2^Facultad de Educación, Universidad de Barcelona, Barcelona, Spain; ^3^Faculty of Education and Social Sciences, Universidad Andres Bello, Santiago, Chile; ^4^Department of Physical Education, Sports, and Recreation, Universidad de La Frontera, Temuco, Chile; ^5^Observatorio de Ciencias de la Actividad Física, Departamento de Ciencias de la Actividad Física, Universidad de Playa Ancha, Valparaíso, Chile; ^6^Instituto Nacional del Fútbol, Deporte y Actividad Física (INAF), Santiago, Chile; ^7^Escuela de Kinesiología, Facultad de Ciencias de la Salud, Universidad Católica Silva Henríquez, Santiago, Chile; ^8^Doctorado en Ciencias de la actividad física y deportes. Universidad de León, Leon, Spain; ^9^Instituto de Literatura y Ciencias del Lenguaje, Pontificia Universidad Católica de Valparaíso; Millennium Nucleus for the Science of Learning (MiNSoL), Valparaiso, Chile; ^10^Escuela de Kinesiología, Facultad de Salud y Odontología, Universidad Diego Portales, Santiago, Chile; ^11^Escuela de Kinesiología, Universidad Autónoma de Chile, Santiago, Chile

**Keywords:** children, physical education, exercise, mental health, academic success

## Abstract

**Objective:**

To determine how cognitive domains mediate the link between fitness components, their global score (GFS), and adolescents’ academic achievement (ACA) across various school subjects.

**Methods:**

In this study, 1,296 adolescents aged 10–14 participated. GFS was computed by three fitness components (strength, muscular, and cardiorespiratory fitness) through the ALPHA-fitness test battery. ACA was determined by five school subjects (Language, English, Mathematics, Science, and History) and two academic scores (a) “Academic Average” (five subjects) and (b) “Academic-PISA” (Language, Mathematics, and Science). A principal component analysis was performed to establish four factors (working memory [WM], cognitive flexibility [*CF*], inhibitory control [IC], and fluid reasoning [FR]). A parallel mediation approach was implemented with 5,000 bootstrapped samples controlled for sex, maturity, central obesity, having breakfast before cognitive tasks, schools, and school vulnerability. Total, direct, indirect effects, and mediation percentages were estimated.

**Results:**

Overall, the finding showed a full parallel mediation effect for Language (92.5%) and English (53.9%), while a partial mediation for Mathematics (43.0%), Science (43.8%), History (45.9%), “Academic Average” (50.6%), and “Academic-PISA” (51.5%). In particular, WM, IC, and FR mediated all school subjects except mathematics, where IC was not significant. *CF* has not mediated any relationship between GF and academic performance.

**Conclusion:**

This study underscores the pivotal role of cognitive domains, specifically WM, IC, and FR, in mediating the link between physical fitness and academic performance in adolescents. These insights have relevant implications for educational and public health policies.

## Introduction

Education is a crucial topic in today’s world since nations’ development depends on it, particularly in unequal world regions like Latin America (Ferreira and Gignoux, 2011). Thus, a pivotal government task is to design and implement novel, cost-effective strategies for education improvement (Barnett, 2008). In this sense, academic achievement (ACA) is one of several educational indicators that emerge to bridge social, economic, and health gaps (Daude and Robano, 2015; Hahn and Truman, 2015). Unfortunately, the COVID-19 pandemic worsened ACA globally due to children’s depression, anxiety, decreased self-esteem, physical activity, and increased sedentary behaviors, which exacerbated educational inequalities (Runacres et al., 2021; Barbosa-Camacho et al., 2022). However, improving physical fitness seems to be a novel strategy for influencing ACA positively by enhancing physical and mental health and reducing cognitive gaps related to social vulnerability (Donnelly et al., 2016; Cristi-Montero et al., 2021).

Robust evidence points to a positive association between physical fitness (mainly cardiorespiratory fitness [CRF]) and ACA (especially in mathematics and English or Spanish as a first language) (Álvarez-Bueno et al., 2020). Besides, evidence also indicates that improving physical fitness can enhance executive functions, especially working memory, inhibitory control, and fluid reasoning (Donnelly et al., 2016; St Laurent et al., 2021). Executive functions refer to a family of top-down mental processes and there is general agreement that there are three core functions; working memory, which enables us to retain information, inhibitory control, which helps suppress automatic responses to stimuli, and cognitive flexibility, which allows us to shift attention between different aspects of a task or problem (Diamond, 2013). From these, high-order executive functions are built such as fluid reasoning and, is the ability to reason, problem solve, and to see patterns or relations among items (Diamond, 2013). All these cognitive abilities play a crucial role in overall cognitive performance and ACA (Ardila, 2008; Diamond, 2013; Blair, 2017).

Hence, adequate cognitive performance (CP) at school age is crucial due to the fact that it is a strong predictor not only ACA but also of health status and economic success in adulthood (Ursache and Noble, 2016). For instance, children with better physical fitness show greater macrostructure in some brain regions, improved microstructure, and increased brain functionality than peers with low physical fitness levels, which have been associated with better CP and ACA (Cadenas-Sanchez et al., 2020). However, all these improvements noted above have been related to neural activity patterns between different executive tasks, which are more sensitive during adolescence (Moisala et al., 2018; Williams et al., 2022). In this sense, there is a scarcity of literature exploring how each cognitive domain could contribute to (i.e., mediating) the relationship between physical fitness and different academic subjects (Visier-Alfonso et al., 2022). Moreover, based on the same principle, each academic subject could be affected by a specific or combination of cognitive domains; hence, expanding this research area beyond mathematics and language could improve our understanding of physical fitness contribution to ACA.

Through a parallel mediation approach, it is possible to establish the mediating role of several mediators, independently and jointly, in the relationship between a predictor and outcome (Kane and Ashbaugh, 2017). This sort of analysis differs from serial mediation because the last assume that one mediator leads the other mediator. In contrast, when mediators are in parallel, we cannot infer causality (Hayes and Andrew, 2013). Indeed, although mediators can be slightly correlated in a parallel approach, they must not create multicollinearity because it may affect the estimation of their partial relationships with the outcome variable (Hayes and Andrew, 2013). In this sense, this study addresses the gap in understanding the specific cognitive domains through which physical fitness impacts academic achievement.

Therefore, the study aimed to establish the parallel mediation role of four cognitive domains (i.e., working memory, fluid intelligence, inhibitory control, and cognitive flexibility) determined by a principal component analysis on the relationship between a global physical fitness score (GFS) and diverse subjects score and average academic achievement in adolescents.

## Methods

This cross-sectional study is part of the Cogni-Action Project conducted between March 2017 and October 2019 (Solis-Urra et al., 2019). This project was retrospectively registered (8/July/2020) with the Research Registry (ID: researchregistry5791) and was approved by the Bioethics and Biosafety Committee of the Ethics Committee of the Pontificia Universidad Católica de Valparaíso (BIOEPUCV-H103-2016). This study was conducted according to the guidelines of the Declaration of Helsinki. Written consent was obtained from the school principal, parents/guardians, and participants before participation.

### Participants

The study enrolled a total of 1,296 adolescent boys and girls (1,1 ratio) aged between 10 and 14 years, corresponding to the 5th to 8th-grade levels, from public, subsidized (i.e., schools receiving government financial support), and private schools in Valparaiso, Chile. Power calculations and total sample size were based on the total enrolment of schoolchildren in the Valparaíso region between 5th and 8th grade (information provided by the Chilean Ministry of Education in 2016). A maximum error of 5%, a confidence interval of 99%, a heterogeneity of 50%, and attrition of 20% were considered based in general lineal, multilevel random-effects models and mediation analysis. Therefore, a total of 797 participants was necessary to reach a representative sample size (Solis-Urra et al., 2019).

### Measurements

Participants were evaluated at school in two 4-h sessions, separated by 8 days. In the first session, body weight, height, waist circumference, and a complete cognitive battery were assessed. The second session evaluated physical fitness through the well-documented ALPHA-fitness test battery (Ruiz et al., 2011). The measurements were performed by trained instructors from our research team. ACA variables were obtained from each student’s school.

### Cardiorespiratory fitness

Cardiorespiratory fitness (CRF) was assessed with the 20 m shuttle run test, which was performed at the end of the assessment session (Ruiz et al., 2011). It was completed in groups of 8–10 participants, with an audible signal indicating the running pace, starting at 8.5 km and increasing by 0.5 km/h per minute. Adolescents participating in the study could withdraw if they experienced perceived fatigue or could not complete the required distance twice. Total time (in seconds) was recorded, and a z-score based on sex and age was created as a standardized CRF score (Tomkinson et al., 2019).

### Muscular fitness

Muscle fitness (MF) was assessed by measuring the upper and lower limbs (Ruiz et al., 2011). The maximum handgrip strength test evaluated the upper limb strength with a dynamometer (Jamar Plus+ Digital Hand Dynamometer, Sammons Preston, Rolyan, Bolingbrook, IL, USA). The dynamometer was previously adjusted for hand size, allowing for 0–90 kg measures, with 0.1 kg precision. The test was performed twice on each hand (alternating both hands), standing with an extended elbow, and the best result of the two measurements was recorded. Then, the score was divided by body weight to create a relative measure of upper limb strength.

Lower extremity strength was evaluated by the standing long jump (SLJ) test. Participants were positioned behind a starting line, feet apart, and jumped as far as possible on the verbal signal, landing with both feet simultaneously. Measurement was performed twice (with at least a one-minute rest between attempts). The greatest distance was recorded in centimeters (Ruiz et al., 2011). Finally, the MF score was calculated based on the sum of the sex- and age-standardized values of handgrip/weight and standing long jump.

### Speed-agility fitness

Speed-Agility fitness (SAF) was assessed using the 4 × 10 m shuttle run test (Ruiz et al., 2011). To carry out the test, two parallel lines were marked on the ground, 5 m long and 10 m apart. The adolescents had to run as fast as possible, carrying a piece of cloth and dropping it on the next line (about 50 cm from the line). Then they picked up another piece of cloth and repeated this sequence thrice. This test was performed twice, and the lowest time was recorded. The time was multiplied by −1, so a higher score indicated better performance. Finally, a *z*-score was created based on gender and age as a standardized SAF score.

### Global fitness score

Global fitness score (GFS) was computed by three fitness components (strength, muscular, and cardiorespiratory fitness) through the ALPHA-fitness test battery (Solis-Urra et al., 2021).

### Academic achievement

ACA was determined by five academic scores (Language, English, Mathematics, Science, and History) and additionally was calculated the “Academic Average” (five subjects) and the “Academic-PISA” (Language, Mathematics, and Science). Final school grades were obtained from official records (semester average) of each school. Grades in Chile are scored between 1 (minimum) and 7 (maximum), in which a grade of four indicates pass.

### Cognitive functioning

The NeuroCognitive Performance Test (NCPT) from Lumos Labs, Inc. (Morrison et al., 2015) assessed cognitive functioning. This test was conducted in groups of 25 students, each with a laptop. This test is a brief, repeatable, web-based platform to measure several cognitive domains, “Trail Making A and B” assessing attention, cognitive flexibility, and processing speed; the “Forward Memory Span” and the “Reverse Memory Span” evaluating short-term visual memory and working memory; the “Go/No-Go” test assessing inhibitory control and processing speed; the “Balance Scale” indicating quantitative and analogical reasoning; the “Digit Symbol Coding” valuing processing speed; and finally, the “Progressive Matrices” assessing problem-solving and reasoning/intelligence (Morrison et al., 2015). Each test was scaled following a normal inverse transformation of the percentile rank and summed, obtaining a global cognitive score (Solis-Urra et al., 2021).

### Covariates

Six covariates (sex, maturity, central obesity, breakfast consumption before cognitive tasks, school type, and school vulnerability) were included in the analysis due to their relevance to the outcome. Sex and maturation are relevant factors in cognitive performance and brain development (Stillman et al., 2020). Maturity was calculated according to the peak height velocity (PHV), subtracting the PHV age from the chronological age (Mirwald et al., 2002). Differences among years were established as a maturity offset value (Moore et al., 2015). Fat distribution, specifically central obesity, has been identified as an important mediating factor in cognitive performance (Hernández-Jaña et al., 2021). The central adiposity indicator (i.e., WHtR) was obtained by measuring waist circumference with an inextensible tape (Lufkin, Apex, NC, USA). Finally, the waist-to-height ratio (WHtR) was obtained by dividing the waist circumference by the subject’s height, both in centimeters (Arnaiz et al., 2010). Eating breakfast before performing a cognitive task and the quality of breakfast are also important factors in cognitive performance, with previous evidence indicating that children who eat breakfast and, specifically, higher quality breakfasts perform better cognitively than those who do not (Peña-Jorquera et al., 2021). On the other hand, the type of school (public, subsidized, or private) and the school vulnerability index (SVI) account for the socioeconomic level, health status, and physical and emotional well-being of pupils and their families (Lemes et al., 2021). In the Chilean context, the government created a method to measure SVI with a score of 0–100, whereas private schools have 0 (López et al., 2017).

### Statistical analysis

Firstly, the database was imputed by the decision tree method implemented in R (MissForest package) (Stekhoven and Bühlmann, 2012). This random forest imputation algorithm is used for mixed data types (numerical or categorical variables); neither pre-processing nor assumptions (i.e., parametric) are required and present a high predictive power. Initially, analyses were performed with 841 subjects (who had data in all variables), and after imputation, the entire study sample size was considered (n = 1,296). Missing data were observed only in GFS (24.5%), breakfast consumption (13.6%), PHV (1.3%), WHtR (3.6%), and academic subjects, ranging between 1.5 and 2.2%. Table 1 shows the *p*-values comparison between sex using imputed and original datasets.

**Table 1:** Adolescents’ characteristics.

**Variables**	**All** **(*n* = 1,296)**	**Boys** **(*n* = 648)**	**Girls** **(*n* = 648)**	***P*-value original**	***P*-value imputed**
Age (y)	11.9 ± 1.2	11.8 ± 1.2	11.9 ± 1.2	0.089	0.089
Peak high velocity	−0.41 ± 1.3	−1.17 ± 1.0	0.34 ± 1.0	**<0.000**	**<0.000**
Waist-to-height ratio				**0.041**	**0.048**
<0.5	1,025 (79.1%)	498 (76.8%)	527 (81.3%)		
>0.5	271 (20.9%)	150 (23.1%)	121 (18.6%)		
Having breakfast*				**<0.000**	**<0.000**
Yes	373 (28.8%)	155 (23.9%)	218 (33.6%)		
No	923 (71.2%)	493 (76.1%)	430 (66.3%)		
**School vulnerability index**				**0.038**	**0.038**
Public	326 (25.2%)	144 (22.2%)	182 (28.2%)		
Subsidized	360 (27.8%)	181 (27.9%)	179 (27.6%)		
Private	610 (47.1%)	323 (49.8%)	287 (44.2%)		
**Physical fitness**					
Cardiorespiratory fitness (*z*)	−0.026 ± 0.92	−0.026 ± 0.92	−0.026 ± 0.92	0.999	0.984
Muscular fitness (*z*)	0.020 ± 0.1.6	0.013 ± 0.1.6	0.028 ± 0.1.5	0.919	0.993
Speed-agility fitness (*z*)	0.000 ± 0.92	0.007 ± 0.92	−0.007 ± 0.93	1.00	0.861
Global fitness score	−0.014 ± 2.9	−0.013 ± 2.9	−0.016 ± 2.8	0.951	0.787
**Cognitive tasks**					
Trail-making test A (*p*)	100.0 ± 14.7	100.2 ± 14.6	99.8 ± 14.8	0.592	0.592
Trail-making test B (*p*)	100.0 ± 14.7	98.9 ± 14.7	101.1 ± 14.6	**0.006**	**0.006**
Memory forward (*p*)	100.0 ± 14.4	100.7 ± 14.3	99.3 ± 14.4	0.091	0.091
Memory reverse (*p*)	100.0 ± 14.4	100.2 ± 14.2	99.7 ± 14.5	0.572	0.572
Go-no-Go (*p*)	100.0 ± 14.7	101.7 ± 14.7	98.3 ± 14.6	**<0.000**	**<0.000**
Scale balance (*p*)	100.0 ± 14.5	99.6 ± 14.3	100.5 ± 14.7	0.266	0.266
Digit coding symbol (*p*)	100.0 ± 14.7	99.1 ± 15.0	100.9 ± 14.3	**0.030**	**0.030**
Progressive matrices (*p*)	100.0 ± 14.3	98.6 ± 14.0	101.5 ± 14.4	**<0.000**	**<0.000**
**Academic achievement**					
Language (*s*)	5.40 ± 0.8	5.27 ± 0.8	5.54 ± 0.8	**<0.000**	**<0.000**
Mathematics (*s*)	5.35 ± 1.0	5.31 ± 1.0	5.40 ± 1.0	0.067	0.061
Science (*s*)	5.45 ± 0.8	5.32 ± 0.8	5.57 ± 0.8	**<0.000**	**<0.000**
English (*s*)	5.62 ± 0.9	5.51 ± 0.9	5.74 ± 0.9	**<0.000**	**<0.000**
History (*s*)	5.45 ± 0.8	5.40 ± 0.8	5.53 ± 0.8	**<0.000**	**<0.000**
Academic average	5.45 ± 0.7	5.35 ± 0.7	5.56 ± 0.7	**<0.000**	**<0.000**
Academic-PISA average	5.40 ± 0.8	5.30 ± 0.8	5.50 ± 0.8	**<0.000**	**<0.000**

In bold significant *p*-values.*Breakfast consumption before the cognitive task; y, years; z, *z*-score; p, percentile; s, score; Academic achievement Chilean scale up to 7.0 score; Academic average: 5 subjects; Academic-PISA average: Language, Mathematics, and Science average.

Secondly, a principal component analysis was conducted to identify and establish four factors: working memory (WM), cognitive flexibility (*CF*), inhibitory control (IC), and fluid reasoning (FR). These factors were determined based on the specific characteristics of our cognitive tasks and existing literature, considering the sample size of our previous publications (Solis-Urra et al., 2021). Thus, four fixed components were established using the Varimax rotation, explaining 71.2% of the variance (WM = 23.3%, *CF* = 18.1%, IC = 16.8%, and FR = 13.0%). The assumption check for Bartlett’s Test of sphericity was significant (*p* < 0.001). Analyses were performed using JAMOVI based on the ‘psych’ Package for R (Revelle, 2019).

Descriptive statistics are shown as mean and standard deviation, or frequency and percentages (Table 1). Parametric tests (*t*-student, chi-square test, correlations, and parallel mediations) were used to conduct analyses, as indicated by the central limit theorem for sample sizes over 500 participants (Lumley et al., 2002). Parallelly, normality distribution was checked visually by a Q-Q plot (quantile-quantile plot). No interaction between sex*fitness (*p* = 0.357) and age*fitness (*p* = 0.494) was observed; thereby, all analyses are presented together for boys and girls and adolescents between 10 and 14 years old. Overall, for all analyses, the significance level was set at *p* < 0.05.

**Figure 1:**
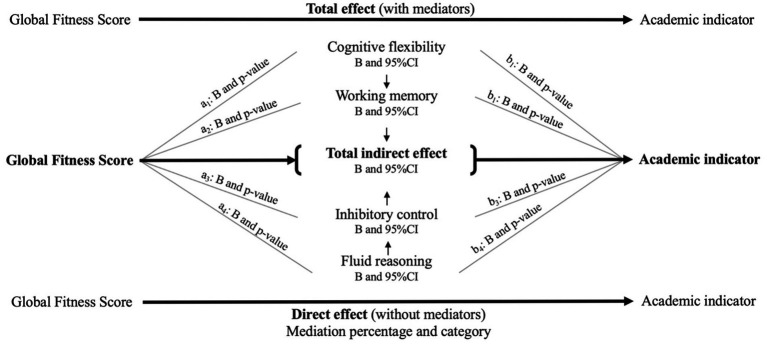
Parallel mediation model.

The mediation approach is presented in Figure 1. A parallel mediation was performed considering GFS as the predictor, all four cognitive domains as mediators (WM, *CF*, IC, and FR), and diverse academic achievements as outcomes. The general mediation model was structured as follows: equation (a) consisted of the predictor (GFS) by the mediators (four cognitive domains); equation (b) mediators by the academic outcomes; equation (c) the predictor by the outcomes (Total Effect); and finally, equation (c’) consisted of predictor and mediators by the outcomes (Direct effect). Mediation was established on the basis of indirect effect. The indirect effect was estimated by a*b equations. Possible bias was reduced by adjusting analyses to relevant covariates such as sex, PHV, WHtR, breakfast consumption, school type, and SVI.

#### Parallel mediation model

The arrows between cognitive domains indicate that they contribute to the total indirect effect. For a detailed findings description, see the Supplementary Figures.

To evaluate the mediation effect, bootstrapping with 5,000 samples, linear regression analysis was performed through PROCESS SPSS script (Hayes, 2017). The indirect effect was considered significant if zero was outside the 95% confidence interval (Hayes and Rockwood, 2017). The percentage of mediation was estimated as 1- (equation c’/equation c). Although using “full/partial” to describe or categorize mediations is discouraged (Hayes and Rockwood, 2017), we employed them due to our substantial sample size (statistical reason) and mainly to improve common reader understanding. A complete mediation occurs when the “Total effect” is statistically significant and the “Direct effect” loses significance after including the mediator; however, in partial mediation, the “Direct effect” does not lose significance. While we aim to ensure reader comprehension, researchers must note that the mediation might not fully capture the phenomenon’s complexity studied.

## Results

A gender-balanced sample of adolescents (50% male and 50% female) aged 10–14 participated in this study. Overall, there were diverse significant differences in variables between boys and girls; however, no interaction by sex was detected. No difference was observed between the imputed and non-imputed datasets. Table 1 shows a complete description of adolescents’ characteristics.

To examine the associations among global fitness scores, cognitive domains, and specific school subjects (Language, Mathematics, History, English, and Science), a correlation matrix was generated. The results of the correlation matrix are presented in Table 2. It is important to note that the correlations between cognitive components are zero, as they are derived from a principal component analysis where each generated component is independent of the others.

**Table 2:** Zero-order correlation matrix between all study variables.

	**a**	**b**	**c**	**d**	**e**	**f**	**g**	**h**	**i**
a. Global fitness Score	—								
b. Cognitive flexibility	**0.103**	—							
c. Working memory	**0.126**	—	—						
d. Inhibitory control	**0.121**	—	—	—					
e. Fluent reasoning	**0.104**	—	—	—	—				
f. Language	**0.093**	**0.209**	**0.104**	**0.229**	**0.129**	—			
g. Mathematics	**0.161**	**0.216**	**0.168**	**0.286**	**0.105**	**0.660**	—		
h. Science	**0.144**	**0.189**	**0.104**	**0.240**	**0.164**	**0.655**	**0.688**	—	
i. English	**0.134**	**0.279**	**0.076**	**0.223**	**0.141**	**0.594**	**0.532**	**0.598**	—
j. History	**0.159**	**0.236**	**0.126**	**0.232**	**0.137**	**0.682**	**0.634**	**0.636**	**0.523**

In bold significant values (*r*). The four cognitive domains must not correlate (equal to zero) because they come from the principal component analysis. Supplementary Table S1 shows a correlation matrix with all eight cognitive tasks.

**Figure 2:**
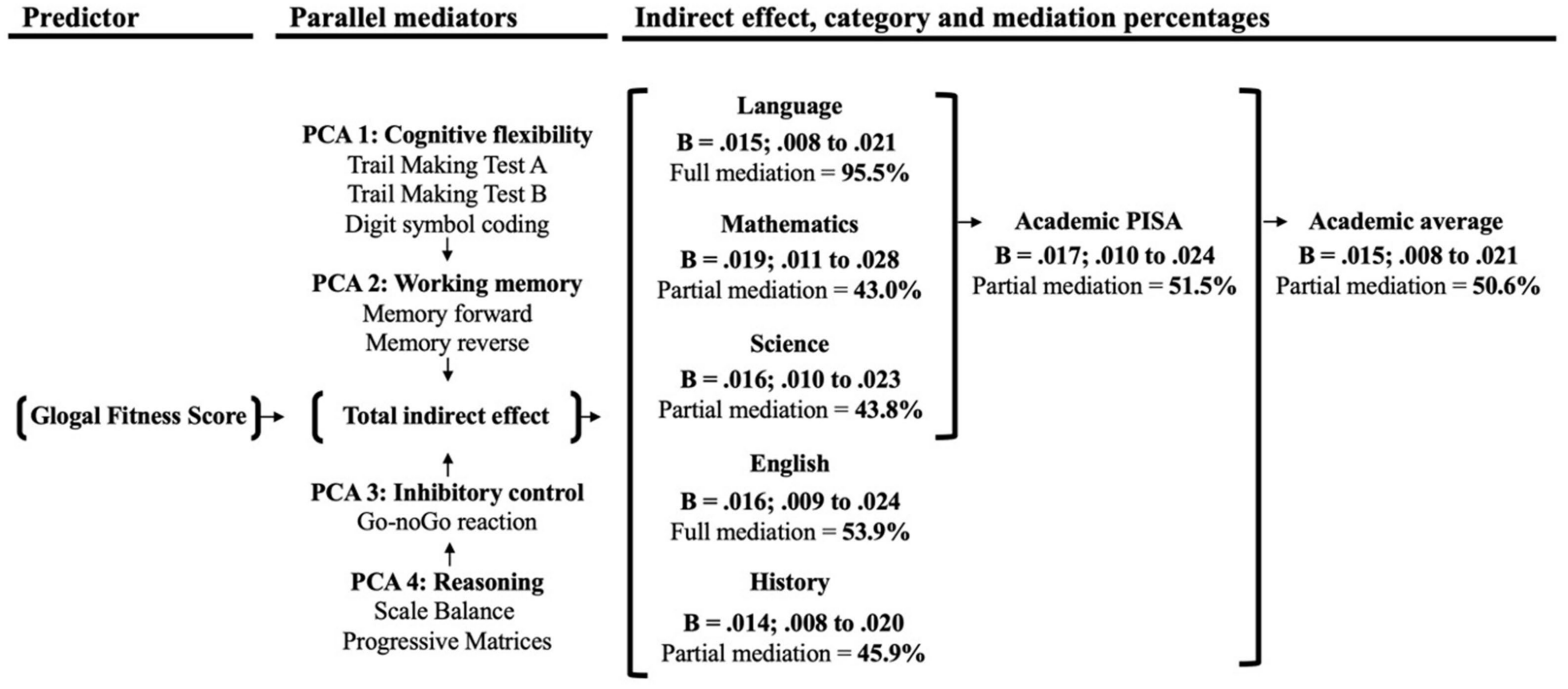
Summary of all mediations addressing in this study.

The results presented in Figure 2 provide a comprehensive overview of the mediations examined in this study. For a detailed description of the findings, including estimates (B non-standardized), 95% confidence intervals (CI), and *p*-values for the total, indirect, and direct effects, please refer to the Supplementary Figures.

Overall, when considering each academic score, our results revealed a full mediation for Language and English, while Mathematics, Science, and History exhibited partial mediation. Additionally, partial mediations were observed for both average scores, namely Academic-PISA and Academic average.

Specifically, for Language and English, the total mediation effects accounted for 92.5% (indirect effect, *B* = 0.015; CI [0.008, 0.021]) and 53.9% (indirect effect, *B* = 0.016; CI [0.009, 0.024]), respectively. Both academic scores demonstrated significant indirect effects on working memory, inhibitory control, and fluid reasoning.

Regarding Mathematics, Science, and History, the partial mediation effects were 43.0% (indirect effect, *B* = 0.019; CI [0.011, 0.018]), 43.8% (indirect effect, *B* = 0.016; CI [0.010, 0.023]), and 45.9% (indirect effect, *B* = 0.014; CI [0.008, 0.020]), respectively. In the case of mathematics, both working memory and fluid reasoning mediated the association with the global fitness score. However, for Science and History, working memory, inhibitory control, and fluid reasoning demonstrated significant indirect effects.

Furthermore, both Academic Average and Academic-PISA exhibited partial mediations (50.6%, *B* = 0.015; CI [0.008, 0.021], and 51.5%, *B* = 0.017; CI [0.010, 0.024], respectively), with significant indirect effects observed for working memory, inhibitory control, and fluid reasoning. No mediation effect was found for cognitive flexibility in any of the analyses.

Summary of all mediations addressing in this study. The arrows *between cognitive domains* indicate that they contribute to the total indirect effect. PCA: Principal component analysis factors and its cognitive tasks. The model was adjusted for age, PHV, WHtR, breakfast consumption, school type, and SVI.

## Discussion

The present study aimed to establish the parallel mediation role of four cognitive domains (i.e., working memory, fluid reasoning, inhibitory control, and cognitive flexibility) in the relationship between a global fitness score and various subject scores and average academic performance in adolescents. In this regard, our results show partial to full mediation of various cognitive domains in this relationship.

On the one hand for the association of GFS and ACA, several studies have shown a possible association especially of cardiorespiratory fitness on academic performance in language, mathematics, and science (Mullender-Wijnsma et al., 2016; McLoughlin et al., 2020; Pate et al., 2020). This evidence is consistent with our results in mathematics (43.0%), science (43.8%), history (45.9%), academic average (50.6%), and academic-PISA (51.5%), where we found a partial mediation of the cognitive domains in the GFS-ACA relationship so that improving fitness could positively influence these subjects through an improvement in cognitive performance. To the best of our knowledge, results in history are a novelty of this study.

The present results can be explained because executive functions and logical-mathematical thinking are related to the frontal and prefrontal lobes of the brain (Henri-Bhargava et al., 2018; Cristofori et al., 2019). CFR causes increased blood flow and oxygenation in this brain area and improves neuroelectric function, neuronal synopsis density (Hillman et al., 2008), neurotrophins related to neurogenesis, and angiogenesis in the brain (Best, 2010). In fact, the literature indicates that children with better CFR have greater total grey and white matter volume (Cadenas-Sanchez et al., 2020). MF also shows these improvements concerning brain function; this is produced by neuromuscular factors but seems to depend on the type of muscle contraction (Yao et al., 2016; Hernández-Jaña et al., 2021). However, regarding the volume of grey and white matter in the brain, the improvements from MF do not appear to be independent of CFR (Esteban-Cornejo et al., 2017, 2020).

On the other hand, we found complete mediation in Language (Spanish as a first language) and English (as a second language), indicating that while GFS is linked to ACA, its influence is largely mediated by improvements in cognitive abilities rather than a direct effect of physical fitness alone. These results are supported by several studies that obtained similar findings associating fitness with mathematics improvements but not reading skills (Hansen et al., 2014; Mullender-Wijnsma et al., 2016). It is worth mentioning that the cognitive domains that were significant in our mediation model for Language and English (working memory, inhibitory control, and fluid reasoning) were the same as for mathematics, history, science, Academic-PISA, and Academic Average.

The underlying mechanism that may explain the variations in performance between reading and mathematics tasks may be attributed to physiological differences in the brain. While these tasks may activate similar neural networks in terms of brain areas involved, the connectivity and myelination give rise to distinct functions within the neural network (Grotheer et al., 2019). In this sense, at the brain level, the areas related to language are Broca’s and Wernicke’s areas (Ono et al., 2022). Language brain areas first start with the language reception/understanding system (Wernicke’s area) and, second, the language production system (Broca’s area), which extends through the basal ganglia and thalamus (Ardila et al., 2016). In this sense, the evidence indicates that language skills are associated with better cognitive performance (Becker et al., 2016; Tremblay and Dick, 2016). In fact, bi-multilingual skills are associated with multiple cognitive benefits (Bialystok et al., 2006a,b; Poulin-Dubois et al., 2013), especially mental flexibility and inhibitory control (Garbin et al., 2010; Prior and Macwhinney, 2010). This may be due to language switching that develops inhibitory control processes (Bialystok and Barac, 2012). Likewise, these multilingual abilities may cause changes in areas of the brain, especially in the gray matter (Stein et al., 2012; Klein et al., 2014). Future studies should explore these differences at the brain level and in executive functions.

Based on our research findings, it is crucial to recognize that improving academic performance requires focusing on both physical and cognitive aspects. Enhancing overall physical fitness alone is not sufficient; rather, attention must also be given to cognitive performance, particularly executive functions such as working memory, inhibitory control, and fluid reasoning. To effectively implement these findings, it is necessary to establish national policies and guidelines that prioritize integrating physical activity into academic subjects and dedicating specific time for physical activity classes. This includes active breaks, school recess, and active commuting, as well as incorporating physical activities that stimulate cognitive functions directly into classroom lessons. Additionally, providing professional development opportunities for teachers is essential to equip them with the necessary tools for implementation. This comprehensive approach will enable students to achieve academic outcomes while promoting well-being.

### Study strengths and limitations

This study has several strengths that contribute to its robustness, including a large sample of adolescents from a developing country, a comprehensive global fitness indicator, diverse cognitive domains, and multiple school subjects. However, limitations exist, such as the indirect measurement of CFR, the absence of emotional variables, a cross-sectional design precluding causal relationships. Additionally, the absence of individual-level socioeconomic status as a covariate could be a significant confounder. Also, the interpretation of full mediation observed in certain subjects and others not, should be taken with caution, since those unmeasured cognitive functions, may contribute to the relationship between GFS and ACA in other subjects. Therefore, future studies should consider others potential mediators. Despite these limitations, the study provides valuable insights into the association between physical fitness, cognitive domains, and academic achievement in adolescents. Future research can address these limitations to further enhance understanding in this area.

## Conclusion

In conclusion, this study highlights the intricate relationship between adolescents’ physical fitness and academic achievement, mediated by performance in diverse cognitive domains. The findings suggest that the association is not solely attributed to fitness improvements but is instead influenced by the convergence of cognitive functioning and physical fitness. Specifically, the complete mediation effect observed in Language and English subjects underscores the significant role of cognitive performance in shaping the relationship. Consequently, implementing targeted strategies that prioritize enhancing physical fitness among adolescents can impact academic achievements positively, contingent upon concurrent improvements at the cognitive level. These insights accentuate the importance of considering physical and cognitive aspects in interventions to promote adolescent academic outcomes.

In conclusion, this study highlights the intricate relationship between adolescents’ physical fitness and academic achievement, mediated by performance in diverse cognitive domains. Our findings indicate that physical fitness, is significantly associated with academic achievement through its influence on cognitive functions such as working memory, inhibitory control, and fluid reasoning. Specifically, the complete mediation effect observed in Language and English subjects underscores the significant role of cognitive performance in shaping the relationship. These results suggest that improvements in academic achievement are not solely due to enhanced physical fitness but also rely heavily on cognitive enhancements. Consequently, our study provides evidence that implementing targeted strategies to enhance physical fitness among adolescents may positively impact academic achievements, provided there are concurrent improvements in cognitive performance. These insights accentuate the importance of integrating both physical and cognitive aspects in interventions to promote adolescent academic outcomes.

## Data availability statement

The raw data supporting the conclusions of this article will be made available by the authors, without undue reservation.

## Ethics statement

The studies involving humans were approved by Ethics Committee of Pontificia Universidad Católica de Valparaíso (BIOEPUCV-H103–2016). The studies were conducted in accordance with the local legislation and institutional requirements. Written informed consent for participation in this study was provided by the participants’ legal guardians/next of kin.

## Author contributions

CC-M: Conceptualization, Data curation, Formal analysis, Funding acquisition, Investigation, Methodology, Project administration, Supervision, Writing – original draft, Writing – review & editing. RM-F: Investigation, Writing – original draft, Writing – review & editing. JE-P: Writing – original draft, Writing – review & editing. AD: Writing – review & editing. JZ-C: Writing – review & editing. NA-F: Writing – review & editing. TR-A: Writing – review & editing. VS-C: Writing – review & editing. RI: Writing – review & editing. KS: Writing – review & editing.
