# Supplementary material for: Substantial parallel mediation contribution by cognitive domains in the relationship between adolescents’ physical fitness and academic achievements: the Cogni-Action Project
Source: Front Psychol. 2024 Jul 10;15:1355434. doi: 10.3389/fpsyg.2024.1355434 (PMC11267617; doi:10.3389/fpsyg.2024.1355434)

**Supplementary material**

**Table S1.** Zero-order correlation matrix between all study variables.

|  | a | b | c | d | e | f | g | h | i | j | k | l | m |
| --- | --- | --- | --- | --- | --- | --- | --- | --- | --- | --- | --- | --- | --- |
| a. GFS |  |  |  |  |  |  |  |  |  |  |  |  |  |
| b. TMTA | **0.099** |  |  |  |  |  |  |  |  |  |  |  |  |
| c. TMTB | **0.071** | **0.431** |  |  |  |  |  |  |  |  |  |  |  |
| d. MEMFO | **0.156** | **0.290** | **0.298** |  |  |  |  |  |  |  |  |  |  |
| e. MEMRE | **0.121** | **0.301** | **0.336** | **0.425** |  |  |  |  |  |  |  |  |  |
| f. Go-noO | **0.124** | **0.285** | **0.206** | **0.174** | **0.191** |  |  |  |  |  |  |  |  |
| g. Balance | **0.146** | **0.270** | **0.350** | **0.271** | **0.285** | **0.240** |  |  |  |  |  |  |  |
| h. Digit | **0.144** | **0.439** | **0.458** | **0.293** | **0.325** | **0.283** | **0.304** |  |  |  |  |  |  |
| i. Matrices | **0.087** | **0.169** | **0.224** | **0.203** | **0.214** | **0.102** | **0.286** | **0.146** |  |  |  |  |  |
| j. Language | **0.070** | **0.226** | **0.231** | **0.150** | **0.221** | **0.171** | **0.305** | **0.233** | **0.185** |  |  |  |  |
| k. English | **0.125** | **0.275** | **0.309** | **0.141** | **0.191** | **0.189** | **0.312** | **0.276** | **0.200** | **0.600** |  |  |  |
| l. Math | **0.130** | **0.234** | **0.287** | **0.220** | **0.258** | **0.165** | **0.368** | **0.277** | **0.210** | **0.671** | **0.523** |  |  |
| m. Science | **0.126** | **0.197** | **0.207** | **0.151** | **0.190** | **0.211** | **0.334** | **0.271** | **0.169** | **0.656** | **0.604** | **0.679** |  |
| n. History | **0.139** | **0.245** | **0.224** | **0.184** | **0.231** | **0.171** | **0.339** | **0.262** | **0.179** | **0.710** | **0.537** | **0.662** | **0.636** |

In bold significant values (r). 1. GFS: Global fitness score; 2. TMTA: Trail making test A; 3. TMTB: Trail making test B; 4. MEMFO: Memory forward; 5. MEMRE: Memory reverse; 6. GO-NOGO: Go-noGo reaction; 7. Balance: Scale balance; 8. Digit: Digit symbol coding; 9. Matrices: Progressive matrices.

**Parallel mediation analysis for all outcomes.**

**Figure S2.** Parallel mediation analysis for English.


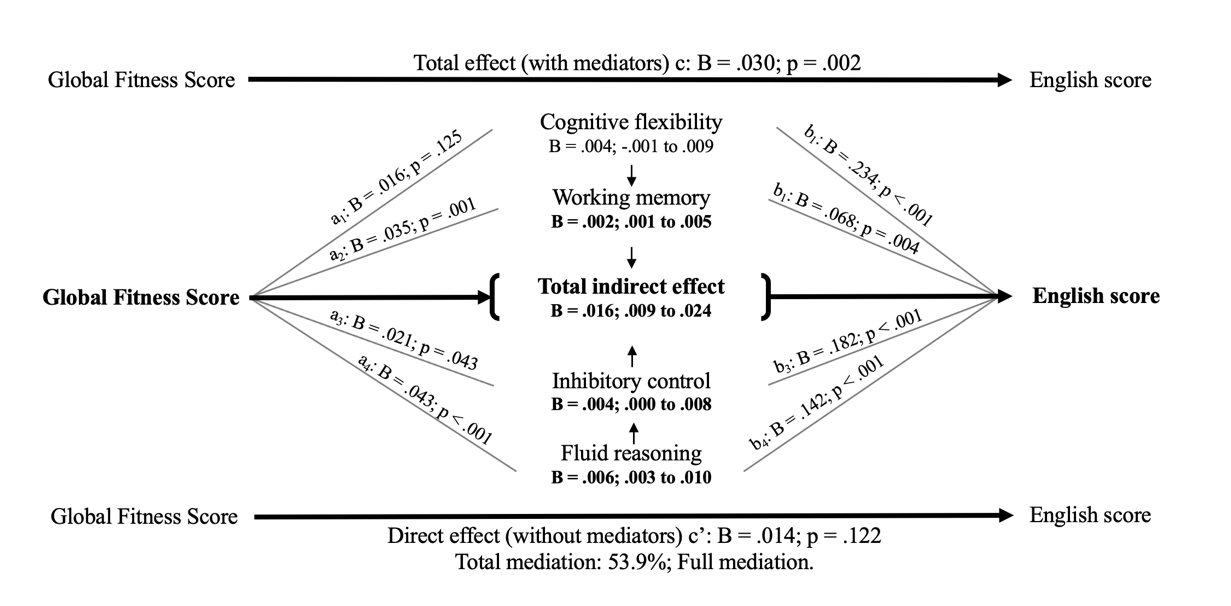


**Figure S3.** Parallel mediation analysis for Language.


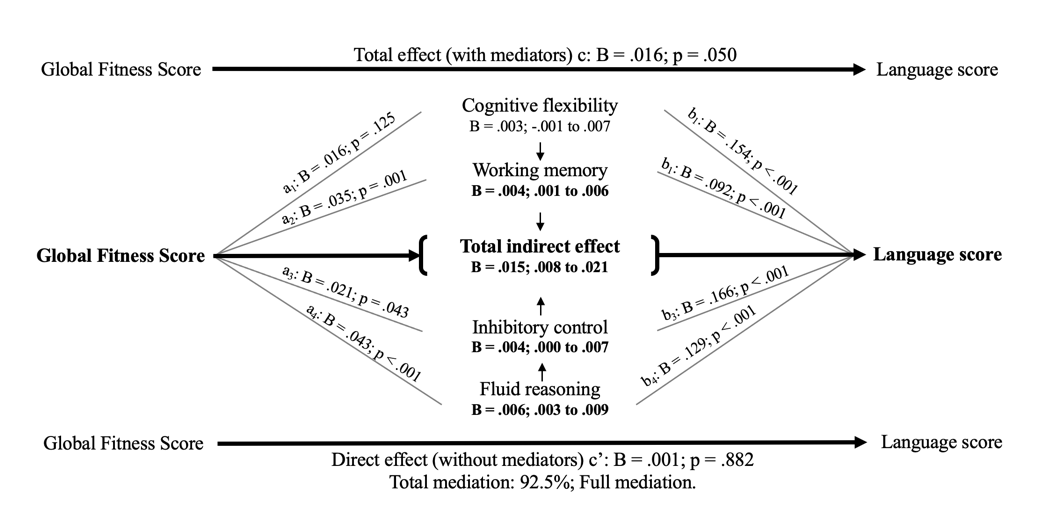


**Figure S4.** Parallel mediation analysis for Mathematics.


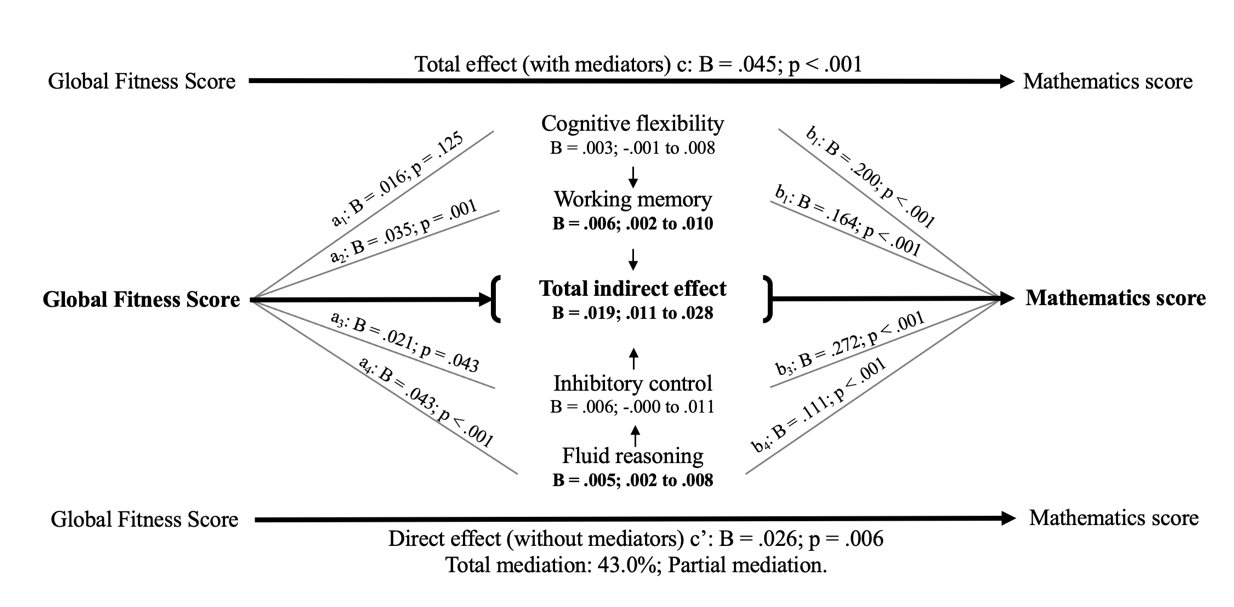


**Figure S5.** Parallel mediation analysis for History.


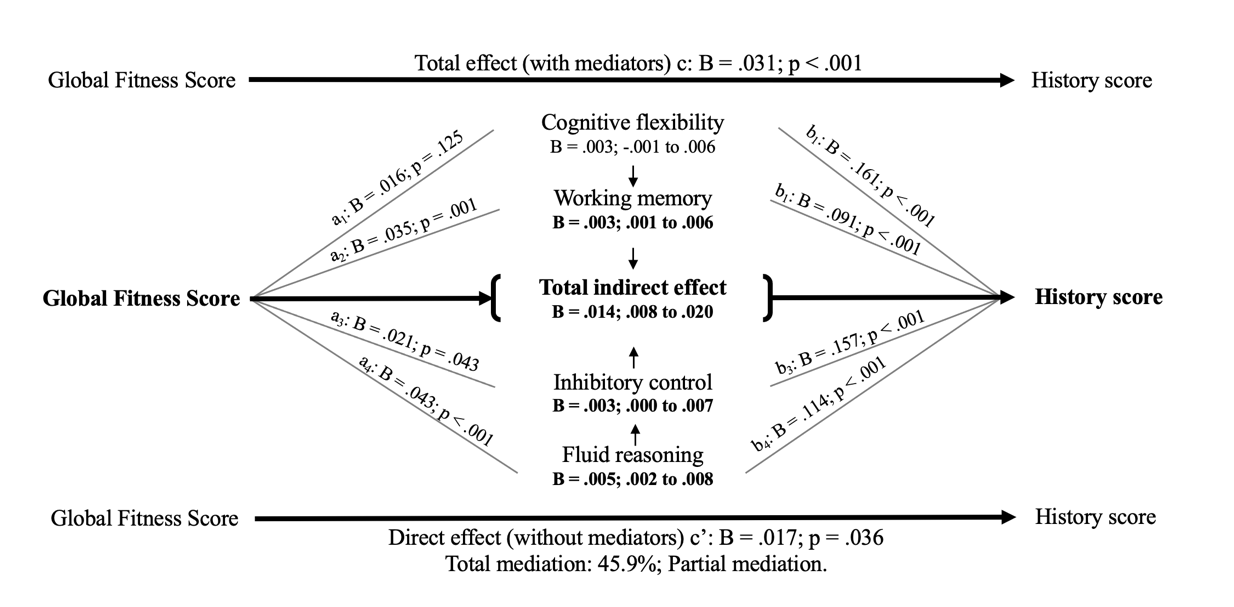


**Figure S6.** Parallel mediation analysis for Science.


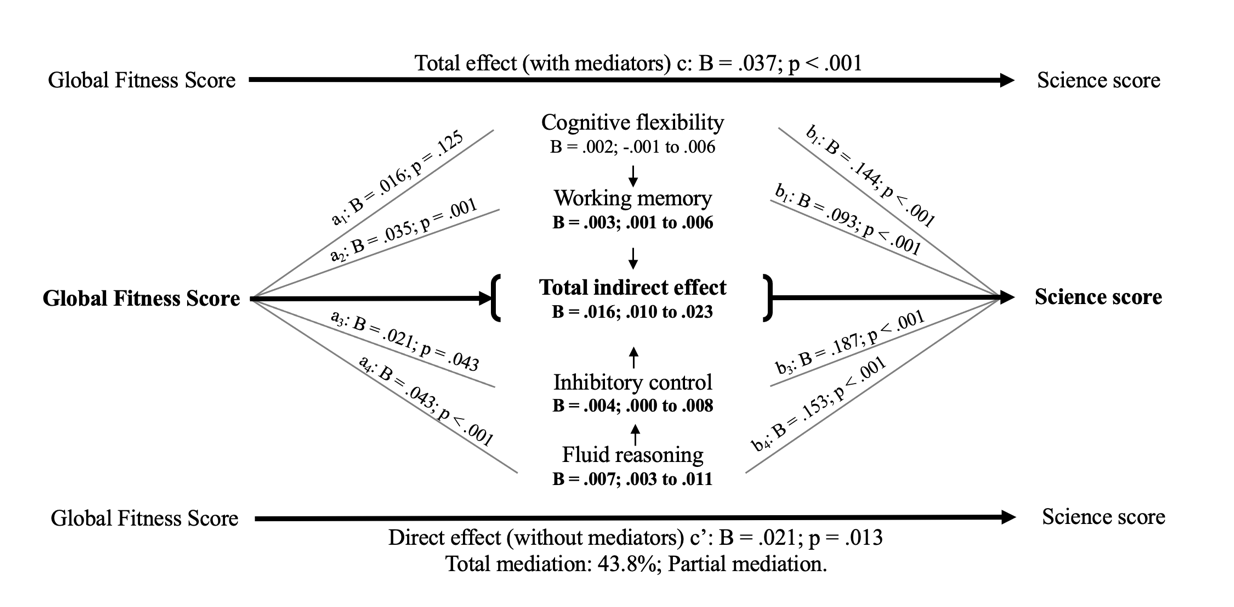


**Figure S7.** Parallel mediation analysis for Academic Average.


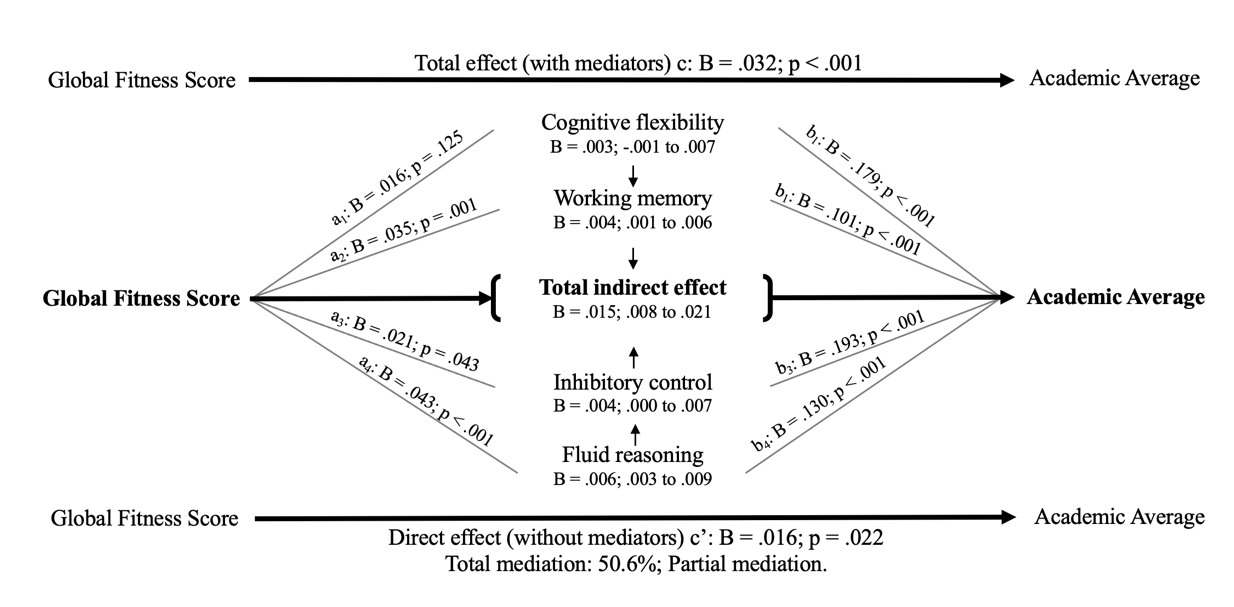


**Figure S8.** Parallel mediation analysis for Academic-PISA.


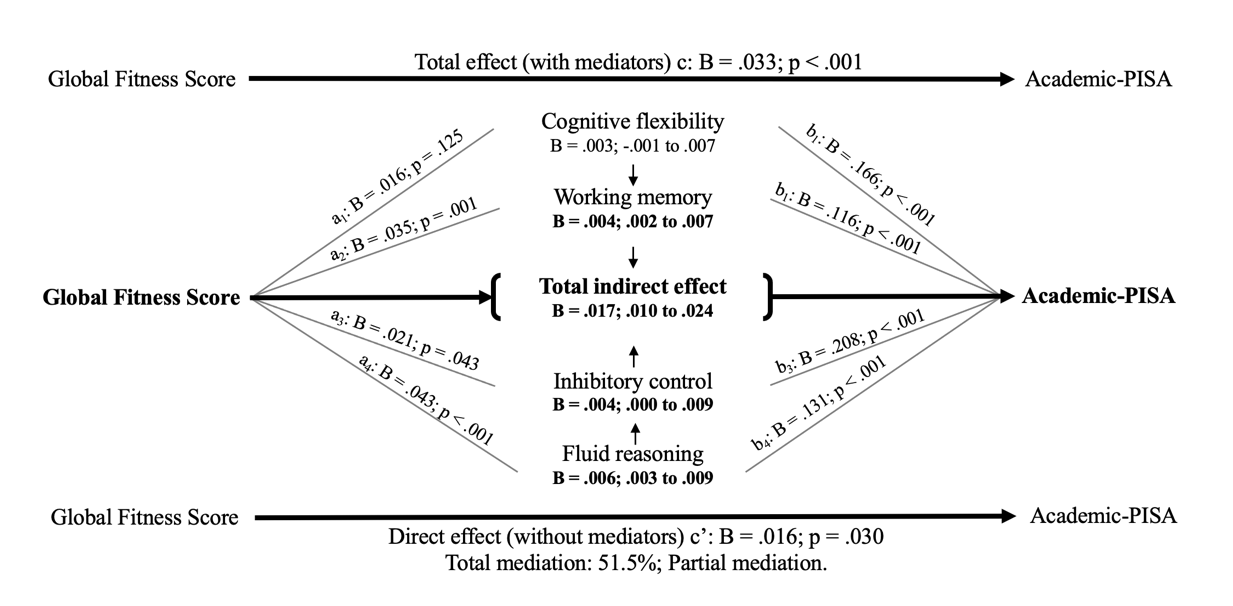

Supplement: Supplementary file 1 [file Data_Sheet_1.docx]
